# Using IMGT unique numbering for IG allotypes and Fc‐engineered variants of effector properties and half‐life of therapeutic antibodies

**DOI:** 10.1111/imr.13399

**Published:** 2024-10-04

**Authors:** Marie‐Paule Lefranc, Gérard Lefranc

**Affiliations:** ^1^ IMGT®, the international ImMunoGeneTics information system® (IMGT), Laboratoire d'ImmunoGénétique Moléculaire (LIGM), Institut de Génétique Humaine (IGH), UMR 9002 Centre National de la Recherche Scientifique (CNRS) Université de Montpellier (UM) Montpellier Cedex 5 France

**Keywords:** allotypes, antibody half‐life, antibody‐dependent cellular cytotoxicity, antibody‐dependent cellular phagocytosis, complement‐dependent cytotoxicity, Fc‐engineered variants

## Abstract

Therapeutic monoclonal antibodies (mAb) are usually of the IgG1, IgG2, and IgG4 classes, and their heavy chains may be modified by amino acid (aa) changes involved in antibody‐dependent cellular cytotoxicity (ADCC), antibody‐dependent cellular phagocytosis (ADCP), complement‐dependent cytotoxicity (CDC), and/or half‐life. Allotypes and Fc‐engineered variants are classified using IMGT/HGNC gene nomenclature (e.g., *Homo sapiens* IGHG1). Allotype names follow the WHO/IMGT nomenclature. IMGT‐engineered variant names use the IMGT nomenclature (e.g., Homsap G1v1), which comprises species and gene name (both abbreviated) followed by the letter v (for variant) and a number. Both allotypes and engineered variants are defined by their aa changes and positions, based on the IMGT unique numbering for C domain, identified in sequence motifs, referred to as IMGT topological motifs, as their limits and length are standardized and correspond to a structural feature (e.g., strand or loop). One hundred twenty‐six variants are displayed with their type, IMGT numbering, Eu‐IMGT positions, motifs before and after changes, and their property and function (effector and half‐life). Three motifs characterize effector variants, CH2 1.6–3, 23‐BC‐41, and the FG loop, whereas three different motifs characterize half‐life variants, two on CH2 13‐AB‐18 and 89–96 with H93, and one on CH3 the FG loop with H115.

## 1 INTRODUCTION

IMGT®, the international ImMunoGeneTics information system®, was founded in 1989 by Marie‐Paule Lefranc (Université de Montpellier (UM), Centre National de la Recherche Scientifique (CNRS), Montpellier, France) in order to manage the huge diversity of the antigen receptors of the adaptive immune responses, immunoglobulins (IG) or antibodies and T cell receptors (TR).^1^ The founding of IMGT marked the birth of immunoinformatics,^1^ a new science at the interface between immunogenetics and bioinformatics.^1^, ^2^ The IMGT nomenclature^3^, ^4^, ^5^, ^6^, ^7^, ^8^, ^9^, ^10^, ^11^ and the IMGT unique numbering^12^, ^13^, ^14^, ^15^, ^16^, ^17^, ^18^, ^19^ with the IMGT Collier de Perles graphical representations^20^, ^21^, ^22^, ^23^, ^24^, ^25^, ^26^ are the two pillars of immunoinformatics.^1^, ^2^, ^27^, ^28^, ^29^, ^30^, ^31^, ^32^, ^33^, ^34^, ^35^, ^36^, ^37^, ^38^, ^39^, ^40^, ^41^, ^42^, ^43^ Indeed, they provide a common language for the genes, sequences, and structures of the IG, TR, and major histocompatibility (MH),^1^, ^2^ based on the IMGT Scientific chart rules,^27^, ^28^, ^29^, ^30^, ^31^, ^32^, ^33^, ^34^, ^35^, ^36^, ^37^, ^38^, ^39^, ^40^, ^41^, ^42^, ^43^ which have been generated from the IMGT‐ONTOLOGY axioms,^1^, ^2^, ^36^, ^44^, ^45^, ^46^, ^47^, ^48^, ^49^, ^50^, ^51^, ^52^ and more particularly from the “CLASSIFICATION” axiom^1^, ^3^, ^4^, ^7^, ^8^, ^52^, ^53^ (for the IMGT nomenclature) and from the “NUMEROTATION” axiom^1^, ^17^, ^18^, ^25^, ^26^ (for the IMGT unique numbering).

The IMGT nomenclature of the IG and TR is extensively used in the analysis by next‐generation sequencing (NGS) or high‐throughput sequencing (HTS) of the adaptive immune receptor repertoires (AIRR) in normal (vaccination) and pathological (e.g., leukemia, lymphoma, myeloma, infectious diseases, and autoimmune diseases) situations, in antibody and T cell receptor analysis of specificity and structures, in therapeutic antibody humanization and engineering, and in the description of the IG and TR genes and alleles in loci of *Homo sapiens* and other jawed vertebrates (*gnathostomata*) in genome assemblies.^1^, ^3^, ^4^, ^10^, ^11^, ^30^, ^40^, ^41^, ^53^

The IMGT unique numbering has been defined for the variable (V)^14^ and constant (C)^15^ domains of the IG and TR and for the groove (G)^16^ domains of the MH. It has been extended, respectively, to the V‐like^14^ and C‐like^15^ domains of any protein of the immunoglobulin superfamily (IgSF) defined as containing at least a V‐like or a C‐like domain (e.g., the two or three C‐like domains of the FCGR [FcgammaR]), and to the G‐like^16^ domains of any protein of the MH superfamily (MhSF) defined as containing two groove domains, each one contributing to one half of the platform and to one helix (e.g., G‐like domain of FCGRT [FcRn], FCGRT belongs also to the IgSF by its C‐like domain).^16^

The IgG1 is the most frequent IG subclass currently used in therapeutic antibodies; however, there is an increasing number of IgG2 and IgG4, and a growing interest for IgG3.^3^, ^10^ The natural IgG is a homodimer of a heavy (H)‐gamma chain paired to a light (L) chain.^l0^ The light chain (L‐kappa or L‐lambda) comprises a variable light (VL) domain (V‐kappa or V‐lambda, V‐J‐REGION [description label in capital letters]^51^) and a constant region (C‐REGION) made of a single constant light (CL) domain (C‐kappa or C‐lambda).^1^, ^3^, ^10^ The H‐gamma chain comprises a variable heavy (VH) domain (V‐D‐J‐REGION) and a C‐REGION made of three constant heavy (CH) domains (CH1, CH2, and CH3), with a hinge region between the CH1 and CH2, and a short CHS region of two amino acids (glycine‐lysine) at the C‐terminal end of the CH3 of the secreted IG.^1^, ^3^, ^10^ The C‐REGION of the *Homo sapiens* (Homsap) H‐gamma1, H‐gamma2, H‐gamma3, and H‐gamma4 chains are encoded by the IGHG1, IGHG2, IGHG3, and IGHG4 genes, respectively.^1^, ^3^, ^10^ Each CH domain is encoded by one exon,^15^, ^43^ the hinge region is encoded by one separate exon in IGHG1, IGHG2, and IGHG4, and usually by four exons in IGHG3, although the number of IGHG3 hinge exons may vary from two to five depending on the alleles.^1^, ^3^, ^10^

Therapeutic antibodies may display natural amino acid (aa) polymorphisms on the H‐gamma CH domains (such as allotypes and isoallotypes^54^, ^55^, ^56^, ^57^, ^58^, ^59^, ^60^, ^61^, ^62^, ^63^, ^64^, ^65^, ^66^, ^67^, ^68^).^1^, ^3^, ^10^ Moreover, therapeutic antibodies CH2 and CH3, part of the fragment crystallizable (Fc), may be modified (engineered) by aa changes involved in antibody‐dependent cellular cytotoxicity (ADCC), antibody‐dependent cellular phagocytosis (ADCP), complement‐dependent cytotoxicity (CDC), and/or half‐life.^69^, ^70^, ^71^ Therapeutic antibodies allotypes (and isoallotypes) and Fc‐engineered variants are classified, using the IMGT nomenclature for genes, alleles, and variants^1^, ^3^ and described using the IMGT unique numbering for C domain^15^ which bridge genes, sequences, and structures.

## 2 THE IMGT NOMENCLATURE

### 2.1 IMGT IG and TR genes and interoperability

The first IMGT breakthrough occurred in 1989 at the tenth Human Genome Mapping (HGM10) workshop in New Haven (USA) when the rearranging variable (V), diversity (D), and joining (J) chromosomal DNA genes, involved in the synthesis of the immunoglobulins (IG) and T‐cell receptors (TR) chains, were finally officially recognized as “genes”^72^, ^73^ as well as were the conventional genes. The IMGT nomenclature which comprises the locus, group, subgroup, gene, and allele concepts^1^, ^3^, ^4^, ^5^, ^6^, ^7^, ^8^, ^9^, ^10^, ^11^, ^53^ was officially approved at the international level by the Human Genome Organisation (HUGO) Nomenclature Committee (HGNC)^74^ in 1999. The IMGT *Homo sapiens* IG and TR gene names^3^, ^4^, ^5^, ^6^ were entered in the National Center for Biotechnology Information (NCBI) LocusLink.^75^

The Immunoglobulin FactsBook^3^ and the T‐cell receptor FactsBook,^4^ published in 2001, are the princeps references for the representations of the *Homo sapiens* IG and TR loci and the nomenclature of the IG and TR genes and alleles, built on more than 10 years of work and expertise by the Laboratoire d'ImmunoGénétique Moléculaire (LIGM) (CNRS, UM, France), and prior to the first sequence of the human genome.^76^, ^77^ The Immunoglobulin FactsBook describes the organization of the three *Homo sapiens* IG loci (IGH, IGK, and IGL) and provides the gene and allele names of 203 functional and open reading frame (ORF) IG genes corresponding to 459 alleles, for a total of 837 genomic sequences.^3^ The T‐cell receptor FactsBook describes the organization of the four *Homo sapiens* TR loci (TRA, TRB, TRG, and TRD) and provides the gene and allele names of 168 functional and ORF TR genes.^4^

The IMGT *Mus musculus* IG and TR genes with IMGT reference sequences were provided to HGNC and to the Mouse Genome Database (MGD)^78^ in July 2002. Amino acid (aa) sequences of the human IMGT IG and TR constant genes (e.g., *Homo sapiens* IGHM, IGHG1, and IGHG2) were provided to UniProt in 2008, and those of the human IG variable genes in 2016, with their IMGT/HGNC gene definition (e.g., *Homo sapiens* IGHV1‐2 immunoglobulin heavy variables 1–2).

**FIGURE 1:**
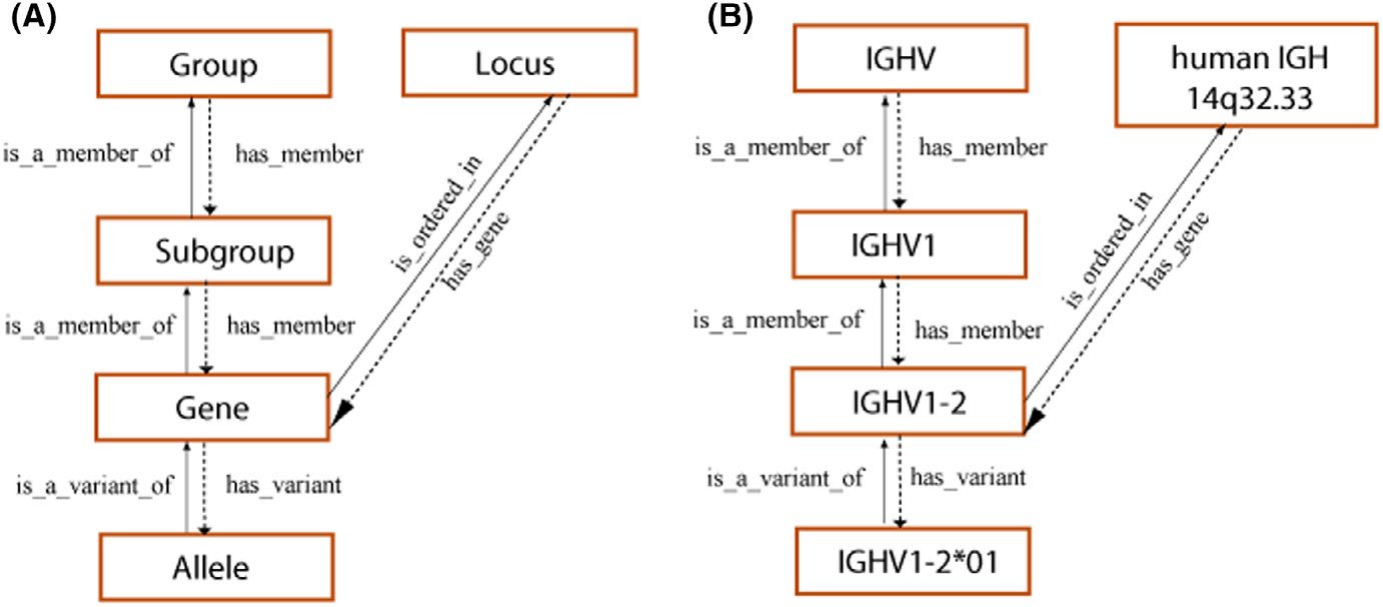
Concepts of classification for IMGT gene and allele nomenclature (CLASSIFICATION axiom).^1^, ^3^, ^4^, ^7^, ^8^, ^52^, ^53^ (A) Hierarchy of the concepts of classification and their relations. The definition of the reciprocal relations between concepts can be read, from one concept to the other, either ascending the hierarchy (solid arrows) or descending the hierarchy (dotted arrows). (B) Examples of concept instances for each concept of classification. The concept instances are associated with an instance of the “Taxon” concept, and more precisely for the “Gene” and “Allele” concepts to an instance of the “Species” concept (here, *Homo sapiens*). The “Locus” concept is a concept of localization (LOCALIZATION axiom).^1^ It is shown with the reciprocal relations to the “Gene” concept. *Source*: With permission from M‐P. Lefranc and G. Lefranc, LIGM, Founders and Authors of IMGT®, the international ImMunoGeneTics information system®, https://www.imgt.org.

HGNC, NCBI Gene, and UniProt have direct links to the *Homo sapiens* IG and TR genes managed in IMGT/GENE‐DB,^79^ the IMGT gene database available online since January 2003. These genes were also integrated in IMGT‐ONTOLOGY on the National Center for Biomedical Ontology (NCBO) BioPortal^80^ and are available, on the same site, in the HGNC ontology and in the National Cancer Institute (NCI) Metathesaurus (NCImetathesaurus). The World Health Organization (WHO)‐International Union of Immunological Societies (IUIS) Nomenclature Committee endorsed the IMGT nomenclature (IMGT‐NC) in 2006 (Figure 1).^7^, ^8^

Since 2008, IMGT gene and allele names have been used in the definition of monoclonal antibodies (mAb) from the WHO International Nonproprietary Name (INN) programme.^9^, ^81^, ^82^ Corresponding aa sequences of therapeutic antibodies, available at WHO INN MedNet,^81^, ^82^ have been entered in IMGT/2Dstructure‐DB^37^, ^38^ and, if three‐dimensional (3D) structures are available from the Research Collaboratory for Structural Bioinformatics (RCSB) Protein Data Bank (PDB), in IMGT/3Dstructure‐DB.^83^, ^84^, ^85^

### 2.2 Alleles, allotypes, and engineered variants definitions

An allele^3^, ^4^, ^52^, ^53^ is a natural variant of a given genomic IG or TR gene (variable, diversity, junction, or constant gene) defined by the nucleotide sequence of its core region (V‐REGION, D‐REGION, J‐REGION, and C‐REGION^51^) in comparison with the sequence of the core region of the gene assigned to the allele *01.^52^ A given aa sequence may therefore correspond to several alleles.^3^, ^4^, ^21^

An allotype by definition is immunogenic.^54^, ^55^ Allotypes of the IG are unique allelic antigenic determinants (or IG “markers”) recognized by specific antibodies (in terms of immunogenicity, they represent B‐cell epitopes).^54^, ^55^ Allotypes correspond to serologically detected aa changes that characterize the polymorphism of an IG chain within a given isotype.^3^, ^10^ Allotypes are shared among individuals within populations.^54^, ^55^ Allotypes identified on the C‐REGION of expressed allelic IG chains, are encoded by alleles of a given C‐GENE.^54^, ^55^ Thus, for example, the G1m allotypes, or gamma1 markers, are expressed on the C‐REGION of the H‐gamma1 chains, and are encoded by alleles of the IGHG1 gene.^54^, ^55^

An isoallotype by definition is immunogenic.^54^, ^55^ Isoallotypes correspond to (a) given amino acid(s) detected serologically in vitro by antibody reagents on the C‐REGION of expressed isotypic chains, but without being polymorphic in these isotypes.^54^, ^55^ Isoallotypes are found on the C‐REGION of the isotypic chains (H‐gamma1, H‐gamma2, H‐gamma3, and H‐gamma‐4), which is encoded by the respective IGHG genes (IGHG1, IGHG2, IGHG3, and IGHG4).^54^, ^55^ In contrast to an allotype which is a “marker” for a given C‐GENE (e.g., G1m1 for IGHG1), an isoallotype is a “non‐marker” and is designated with the letter “n” preceding the name of the antithetical allotype (e.g., nG1m1, present on the IGHG1 chains which are G1m1‐negative and on the other IGHG chains [G2, G3 and G4] which represent different isotypes).^54^, ^55^

The amino acids encoding all current known allotypes (and isoallotypes) have been characterized^54^, ^55^ allowing the current assignment of allotypes to therapeutic antibodies, based on the chain aa sequences^9^, ^56^, ^57^ and by mass spectrometry.^58^, ^59^, ^60^ The original allotype (and isoallotype) identification is performed by a classical reaction of hemagglutination inhibition,^54^, ^55^ requiring antibody reagents,^61^, ^62^, ^63^, ^64^, ^65^, ^66^, ^67^, ^68^ this means that in the absence of antibody reagents, no new allotype (or isoallotype) can be identified, unless predicted serologically.^54^, ^55^, ^58^

Engineered aa variants are localized on the fragment crystallizable (Fc) region of the therapeutic antibodies, that have been engineered to modify the effector properties of the therapeutical antibodies and/or their half‐life.^69^, ^70^ The IMGT nomenclature of Fc‐engineered variants for effector properties and half‐life has recently been implemented.^71^

## 3 THE IMGT UNIQUE NUMBERING

### 3.1 IMGT unique numbering for V domain, C domain, and G domain

The second IMGT breakthrough was to consider the variable and constant domains of the IG and TR, as evolutionary‐related structural aa units, despite their fundamental differences in nucleotide sequences (rearranged V‐(D)‐J region for the IG and TR V domain versus C‐region encoded by one or several exon(s) for the C domain, rearranged complementarity determining region (CDR)3‐IMGT for the IG and TR V domain versus FG loop for the C domain), in number of strands and loops [“9” strands (A to C, C′, C″, D to G) and “3” loops (BC, C′C″, FG) corresponding to the three CDR‐IMGT for the V domain versus “7” strands (A to G) and “2” loops (BC, FG) (no C′ and C″ strands and no C′C″ loop but instead a transversal CD strand) for the C domain)].^1^, ^10^, ^14^, ^15^, ^43^

The IMGT structural approach of the aa sequences of the domains led to the standardized IMGT unique numbering for the V domain^14^ (V‐DOMAIN of the IG and TR and V‐LIKE domain of the other members of the immunoglobulin superfamily [IgSF]) and to the standardized IMGT unique numbering for C domain^15^ (C‐DOMAIN of the IG and TR and C‐LIKE domain of the other members of the IgSF). The concept of unique numbering has been extended similarly to the G domain of the major histocompatibility (MH) proteins of class I (MH1) and of class II (MH2), despite the different chain contribution to the MH groove and platform (one chain, I‐ALPHA, for MH1, and two chains, II‐ALPHA and II‐BETA, for MH2).^1^ This led to the standardized IMGT unique numbering for the G domain^16^ (G‐DOMAIN of the MH1 and MH2 and G‐LIKE domain of the other members of the major histocompatibility superfamily (MhSF)).

**FIGURE 2:**
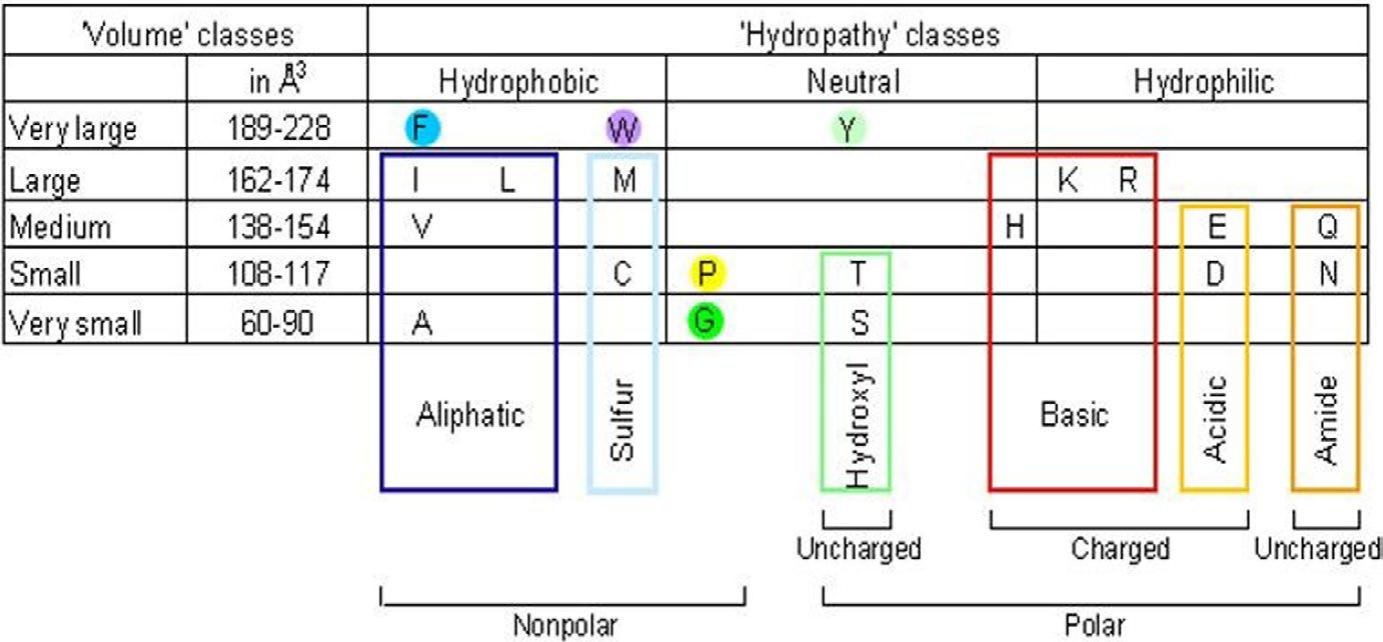
IMGT classes of the 20 common amino acids for the “hydropathy,” “volume,” “chemical characteristics” properties (IDENTIFICATION^50^).^21^ Three “hydropathy” classes (hydrophobic, neutral, and hydrophilic), five “volume” classes in angstrom3 (A3) ([60–90], [108–117], [138–154], [162–174], and [189–228]) and 11 “chemical characteristics” (aliphatic, sulfur, hydroxyl, acidic, amide, basic, F, W, Y, G, and P) classes were defined.^21^ Tyrosine is in the neutral class, although its OH is weakly acidic and polar. Histidine is in the neutral class, although it is weakly basic.^21^ Amino acid one‐letter and three‐letter abbreviations: A (Ala), alanine; C (Cys), cysteine; D (Asp), aspartic acid; E (Glu), glutamic acid; F (Phe), phenylalanine; G (Gly), glycine; H (His), histidine; I (Ileu), isoleucine; K (Lys), lysine; L (Leu), leucine; M (Met), methionine; N (Asn), asparagine; P (Pro), proline; Q (Gln), glutamine; R (Arg), arginine; S (Ser), serine; T (Thr), threonine; V (Val), valine; W (Trp), tryptophan; Y (Tyr), tyrosine. *Source*: With permission from M‐P. Lefranc and G. Lefranc, LIGM, Founders and Authors of IMGT®, the international ImMunoGeneTics information system®, https://www.imgt.org.

The amino acid changes are classified according to the 11 IMGT physicochemical class properties^21^ of the 20 usual amino acids (Figure 2).

### 3.2 C domain characteristics and IMGT Collier de Perles of the IGHG CH

**FIGURE 3:**
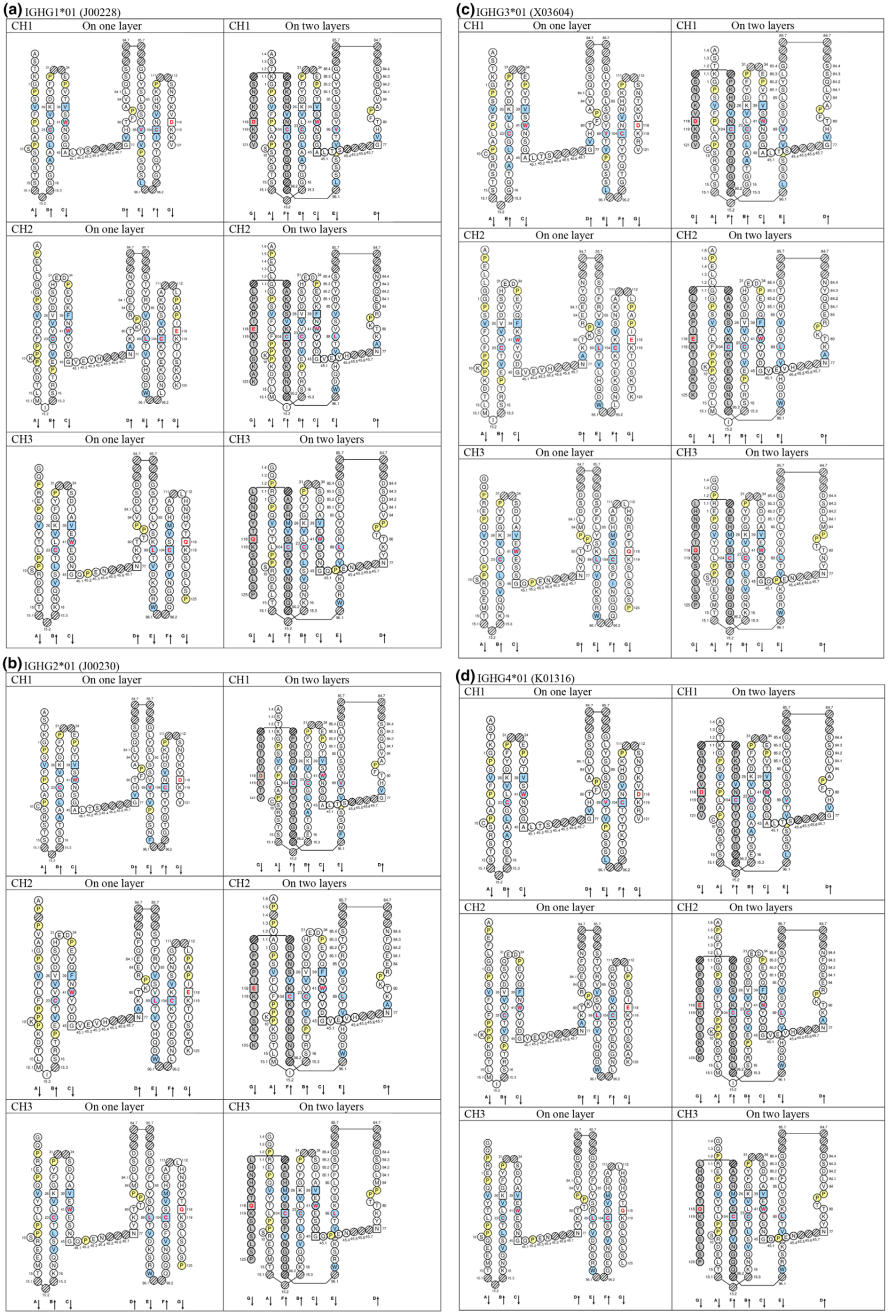
IMGT Colliers de Perles “On one layer” (left column) and “On two layers” (right column) of CH1, CH2, and CH3 encoded by the four *Homo sapiens* IGHG genes.^3^, ^10^, ^22^, ^23^, ^24^, ^25^, ^26^ A, IGHG1*01 (J00228). B, IGHG2*01 (J00230). C, IGHG3*01 (X03604). D, IGHG4*01 (K01316). J00228, J00230, X03604, and K01316 are the GEDI (GenBank/European Nucleotide Archive (ENA)/Database of Japan (DDBJ) and IMGT/LIGM‐DB^1^, ^27^, ^28^, ^29^, ^30^, ^31^, ^32^, ^33^, ^34^, ^35^, ^36^, ^37^) accession numbers of the IMGT/HGNC nucleotide and translation of the IGHG1*01, IGHG2*01, IGHG3*01, and IGHG4*01 IMGT reference sequences.^3^ Arrows indicate the direction of the seven beta strands A to G in 3D structures. IMGT anchors are in square. Hatched circles are unoccupied positions according to the IMGT unique numbering for C domain.^15^ Amino acids are shown in the one‐letter abbreviation (Figure 2). All proline (P) are in yellow. The hydrophobic amino acids (hydropathy index with positive value: I, V, L, F, C, M, A, and tryptophan W)^21^ found at a given position in more than 50% of sequences are shown with a blue background color. Positions with bold letters in red indicate the four conserved aa positions that are common to the V domain and to the C domain: C23 (1st‐CYS), W41 (CONSERVED‐TRP), 89 (hydrophobic), C104 (2nd‐CYS), and the position 118 (diverse in the C domain,^15^ in contrast to the highly conserved and hydrophobic J‐REGION F118/W118 in the V domain^14^). *Source*: With permission from M‐P. Lefranc and G. Lefranc, LIGM, Founders and Authors of IMGT®, the international ImMunoGeneTics information system®, https://www.imgt.org.

A C domain comprises about 90–100 amino acids and is made of seven antiparallel beta strands (A, B, C, D, E, F, and G), linked by beta turns (AB, DE, and EF), a transversal strand (CD) and two loops (BC and FG), and forming a sandwich of two sheets (ABED) (GFC).^15^ A C domain has a topology and a 3D structure similar to that of a V domain^14^ but without the C′ and C″ strands and the C′C″ loop, which are replaced by a transversal CD strand. The lengths of the strands and loops of the C domain are visualized in the IMGT Colliers de Perles,^22^, ^23^, ^24^, ^25^, ^26^ on one layer and two layers. Figure 3 shows the IMGT Colliers de Perles of the three CH domains, CH1, CH2, and CH3, encoded by the four *Homo sapiens* IGHG genes, IGHG1*01, IGHG2*01, IGHG3*01, and IGHG4*01.^3^, ^10^

A C domain^15^ has six IMGT anchors (four of them identical to those of a V domain^14^): positions 26 and 39 (anchors of the BC loop), 45 and 77 (by extension, anchors of the CD transversal strand as there is no C′‐C″ loop in a C domain), and 104 and 118 (anchors of the FG loop) (Figure 3). A C domain has four highly conserved and hydrophobic amino acids which are common to the V domain: cysteine C23 (1st‐CYS), tryptophan W41 (CONSERVED‐TRP), hydrophobic 89, and cysteine C104 (2nd‐CYS).^14^, ^15^ These four amino acids contribute to the two major features shared by the V domain and the C domain: the disulfide bridge (between the two cysteines 23 and 104) and the internal hydrophobic core of the domain (with the side chains of tryptophan W41 and hydrophobic 89).^14^, ^15^

### 3.3 Structural features of the C domain template and of the IGHG CH

The delimitations and the length range of the structural features of the C domain and of the CH1, CH2, and CH3 encoded by the four *Homo sapiens* IGHG genes are shown in Table 1, based on the IMGT unique numbering for C domain.^15^

**TABLE 1:** Structural features of the C domain and of the *Homo sapiens* IGHG CH. A, C domain template,^15^ B, CH1, C, CH2, and D, CH3, of the four *Homo sapiens* IGHG genes (IGHG1, IGHG2, IGHG3, and IGHG4), based on the IMGT unique numbering for C domain.^15^

(A) C domain template^15^			
C domain structural features^a^	IMGT positions^b^	Lengths^c^	Characteristic IMGT Residue@Position^d^
A‐EXTN	1.6–1.1	0–6 (or more)	
A‐STRAND	1–15	15 (14 if gap at 10)	
AB‐TURN	15.1–15.3	0–3	
B‐STRAND	16–26	11	1st‐CYS 23
BC‐LOOP	27–31, 34–38	10 (or less)	
C‐STRAND	39–45	7	CONSERVED‐TRP 41
CD‐STRAND	45.1–45.9	0–9	
D‐STRAND	77–84	8 (or 7 if gap at 82)	
DE‐TURN	84.1–84.7, 85.1–85.7	0–14	
E‐STRAND	85–96	12	Hydrophobic 89
EF‐TURN	96.1–96.2	0–2	
F‐STRAND	97–104	8	2nd‐CYS 104
FG‐LOOP	105–117	13 (or less, or more)	
G‐STRAND	118–128	11 (or less)	

(B) CH1 of the four *Homo sapiens* IGHG			
CH1 structural features^a^	IMGT positions^b^	Lengths^c^	Characteristic IMGT Residue@Position^d^
A‐EXTN	1.4, 1.3, 1.2, 1.1	4	
A‐STRAND	1–15	15	
AB‐TURN	15.1–15.3	0	
B‐STRAND	16–26	11	1st‐CYS 23
BC‐LOOP	27–30, 35–38	8	
C‐STRAND	39–45	7	CONSERVED‐TRP 41
CD‐STRAND	45.1, 45.2, 45.3	3	
D‐STRAND	77–84	8	
DE‐TURN	84.1, 84.2, 84.3, 84.4 85.4, 85.3, 85.2, 85.1	8	
E‐STRAND	85–96	12	Hydrophobic 89
EF‐TURN	96.1–96.2	0	
F‐STRAND	9 8–104	7 (unoccupied 97)	2nd‐CYS 104
FG‐LOOP	105–110, 113–117	11	
G‐STRAND	118–121	4 (instead of 8)	
CH1 length		64 +34 = 98	

(C) CH2 of the four *Homo sapiens* IGHG			
CH2 structural features^a^	IMGT positions^b^	Lengths^c^	Characteristic IMGT Residue@Position^d^
A‐EXTN	1.6, 1.5., * 1.4, 1.3, 1.2, 1.1	6 (*5 in IGHG2)	*1.4 unoccupied in IGHG2
A‐STRAND	1–15	15	
AB‐TURN	15.1, 15.2	2	
B‐STRAND	16–26	11	1st‐CYS 23
BC‐LOOP	27–31, 34–38	10	
C‐STRAND	39–45	7	CONSERVED‐TRP 41
CD‐STRAND	45.1, 45.2, 45.3, 45.4	4	
D‐STRAND	77–84	8	
DE‐TURN	84.1, 84.2, 84.3, 84.4 85.4, 85.3, 85.2, 85.1	8	
E‐STRAND	85–96	12	Hydrophobic 89
EF‐TURN	96.1–96.2	0	
F‐STRAND	97–104	8	2nd‐CYS 104
FG‐LOOP	105–110, 113–117	11	
G‐STRAND	118–125	8	
CH2 length		69 + 41 (* 40 in G2) = 110 (*109 in G2)	

(D) CH3 of the four *Homo sapiens* IGHG			
CH3 structural features^a^	IMGT positions^b^	Lengths^c^	Characteristic IMGT Residue@Position^d^
A‐EXTN	1.4, 1.3, 1.2, 1.1	4	
A‐STRAND	1–15	15	
AB‐TURN	15.1–15.3	0	
B‐STRAND	16–26	11	1st‐CYS 23
BC‐LOOP	27–30, 35–38	8	
C‐STRAND	39–45	7	CONSERVED‐TRP 41
CD‐STRAND	45.1, 45.2, 45.3, 45.4	4	
D‐STRAND	77–84	8	
DE‐TURN	84.1, 84.2, 84.3, 84.4 85.4, 85.3, 85.2, 85.1	8	
E‐STRAND	85–96	12	Hydrophobic 89
EF‐TURN	96.1–96.2	0	
F‐STRAND	97–104	8	2nd‐CYS 104
FG‐LOOP	105–110, 112–117	12	
G‐STRAND	118–125	8	
CH3 length		69 + 36 = 105	

*Note*: In green, IMGT positions and lengths of the A‐extension (A‐extn, A‐EXTN), turns and loops which differ between the CH1, CH2, and CH3 of the *Homo sapiens* IGHG, and in red, unoccupied positions. In blue (Table 1B), IMGT positions and lengths of the F‐strand and G‐strand of CH1, which differ from those of CH2 and CH3. Alternate rows with a blue shade correspond to the seven strands.

*Source*: With permission from M‐P. Lefranc and G. Lefranc, LIGM, Founders and Authors of IMGT®, the international ImMunoGeneTics information system®, https://www.imgt.org.

^a^ IMGT® labels (concepts of description) are written in capital letters (no plural).^51^

^b^ Based on the IMGT unique numbering for C domain.^15^

^c^ In number of amino acids (or codons).

^d^ IMGT Residue@Position is a given residue (usually an amino acid) or a given conserved property amino acid class, at a given position in a domain, based on the IMGT unique numbering.^15^

The IMGT numbering highlights the common features and the specific differences in terms of structure between the three domains CH1, CH2, and CH3 of the *Homo sapiens* IGHG genes. The five strands A, B, C, D, and E are of identical lengths (in number of aa) in the three CH domains of the IGHG genes: A (15 aa), B (11 aa), C (7 aa), D (8 aa), and E (12 aa). The two strands F and G are also of identical lengths: F (8 aa) and G (8 aa) in CH2 and CH3, whereas they are shorter in CH1: F (7 aa) (owing to the unoccupied position 97, near the EF turn) and to the shorter G‐strand (4 aa, instead of 8). The seven strands (contributed by 64 aa for CH1 and 69 aa for CH2 and for CH3) form the conserved structural framework of the three CH domains of the four *Homo sapiens* IGHG genes: 1–15 (A‐strand), 16–26 (B‐strand with C23), 39–45 (C‐strand with W41), 77–84 (D‐strand), 85–96 (E‐strand), 97 (98 in CH1)‐104 (F strand with C104), and 118–125 (121 in CH1) (G‐strand) (Table 1). The IMGT numbering highlights that the length of the “A‐extn, turns and loops” is diverse between the three CH domains of a given IGHG chain, 34 aa for CH1, 41 aa for CH2 (except 40 aa for CH2 of IGHG2), and 36 aa for CH3, in agreement with their different participation in effector properties and half‐life.^71^

In summary, the total length of the CH1 (98 aa) and CH3 (105 aa) domains is identical for the four IGHG genes, whereas the total length of the CH2 is identical for IGHG1, IGHG3 and IGHG4 (110 aa), but differs for IGHG2 (109 aa), the A‐extn 1.4 position being unoccupied (Table 1) owing to a three nucleotides (nt) of the CH2 codon 3.^3^, ^10^ The IMGT genomic approach takes into account the exon splicing sites to delimit the domains and therefore integrates, in the C domain, the A‐strand and A‐extn (functionally important for the effector properties) and the G‐strand, that are often omitted in the structural multiple aa sequence alignments.^17^, ^18^, ^19^, ^43^

### 3.4 Amino acid sequences of the IGHG CH using the IMGT unique numbering

The amino acid sequences of CH1, CH2, and CH3 of *Homo sapiens* IGHG1*01^3^ are displayed in Table 2, using the IMGT unique numbering for C domain.^15^ Correspondence is made to the Eu‐IMGT positions, defined by the IMGT unique numbering for C domain^15^ in the Eu H‐gamma1 CH delimited using the IMGT exon numbering.^3^

**TABLE 2:** IMGT unique numbering of the CH1, CH2, and CH3 of *Homo sapiens* IGHG1*01 (J00228)^3^ and correspondence to the Eu‐IMGT positions^3^, ^15^ (IMGT Scientific chart https://www.imgt.org/IMGTScientificChart/Numbering/Hu_IGHGnber.html).

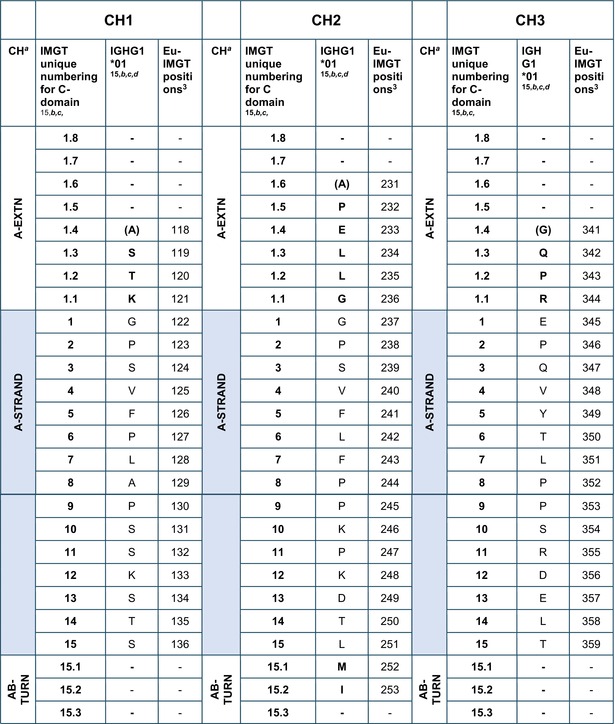
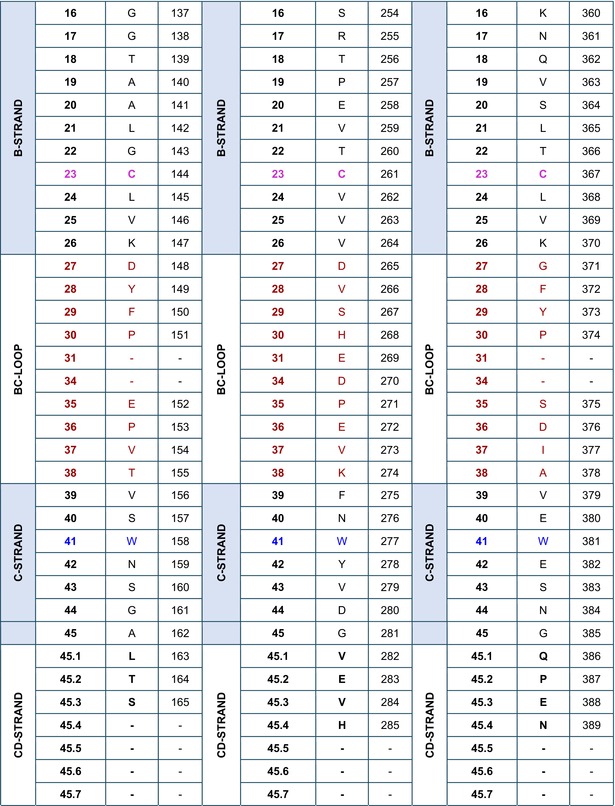
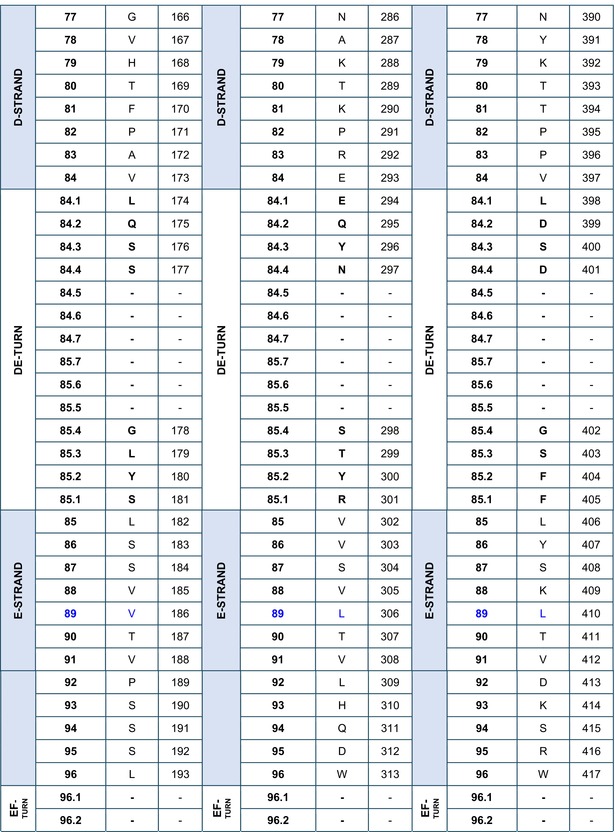
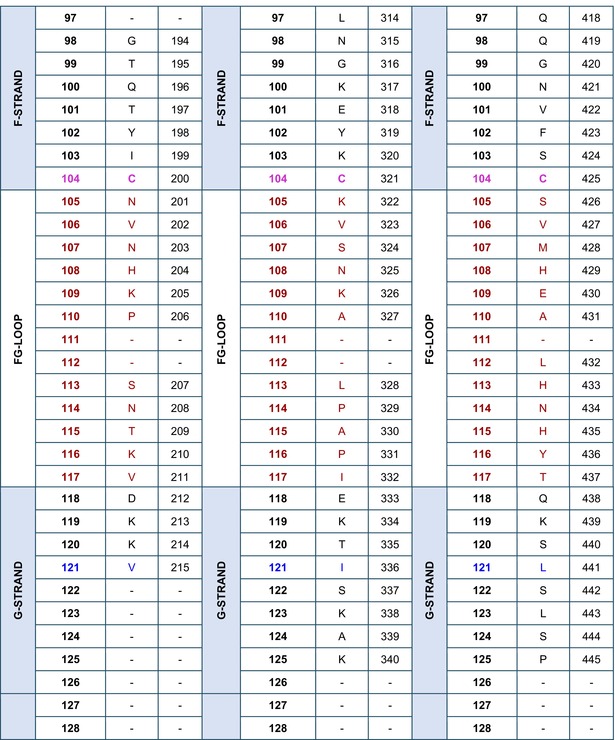

*Source*: With permission from M‐P. Lefranc and G. Lefranc, LIGM, Founders and Authors of IMGT®, the international ImMunoGeneTics information system®, https://www.imgt.org.

^a^ The 14 IMGT labels which describe the CH domains, from the N‐terminal end to the C‐terminal end, are listed from A‐EXTN to G‐STRAND, vertically, for the CH1, CH2, and CH3 domains. The seven strands A, B, C, D, E, F, and G are highlighted with a pale blue background color.

^b^ The BC‐LOOP (27–31, 34–38) and FG‐LOOP (105–117) which are the equivalent of the CDR1‐IMGT (27–38) and CDR3‐IMGT (105–117) of the V‐DOMAIN, respectively, are shown with light brown numbers and letters.

^c^ Amino acids at additional positions with a dot (e.g., 1.4 to 1.1) are in bold. 1st‐CYS C23 and 2nd‐CYS C104 are highlighted in pink bold. W41 and hydrophobic 89 (and 121) are in blue.

^d^ The first aa between parentheses is encoded by a codon which results from the splicing with the donor‐splice of the upstream 5′ exon (https://www.imgt.org/IMGTeducation/Aide‐memoire/_UK/splicing/).^3^, ^10^

The aa sequence of the IMGT CH1 (1.4–121) (98 aa), CH2 (1.6–125) (110 aa), and CH3 (1.4–125) (105 aa) of IGHG1*01 was obtained by translation of the CH1, CH2, and CH3 exons of J00228 (gDNA) (https://www.imgt.org/ligmdb/view?id=J00228), the IMGT reference sequence for the IGHG1*01 allele^3^ (Alignment of alleles: Human IGHG1 https://www.imgt.org/IMGTrepertoire/Proteins/alleles/index.php?species=Homo%20sapiens&group=IGHC&gene=IGHG1). The IGHG1*01 allele encodes the constant region of a H‐gamma1 chain, which expresses the allotypes G1m1,17 (CH1 K120, CH3 D12, and L14) (Table 2, Figure 3A).^54^, ^55^ The translation of the hinge region of 15 aa between the CH1 and CH2 (IMGT H 1–15) and that of the CHS of 2 aa (IMGT CHS 1–2), which are not part of the CH domains, are not shown in Table 2.

### 3.5 IMGT/DomainGapAlign

The IMGT/DomainGapAlign tool is the IMGT tool dedicated to the analysis per domain (e.g., V, C, and G, selected in the interface) of a single aa sequence.^84^, ^86^, ^87^ For “C,” the analysis is performed by alignment of the submitted sequence against the C domain IMGT reference directory (made of sequences annotated using the IMGT nomenclature and the IMGT unique numbering). The results of the analysis are provided with the C domain header.^17^, ^84^, ^86^, ^87^ The IMGT Collier de Perles for C domain (on one layer or two layers)^25^ (Figure 3) can be obtained directly from the IMGT/DomainGapAlign interface^84^, ^86^, ^87^ or using the IMGT/Collier‐de‐Perles tool.^24^

### 3.6 IMGT Protein displays of IGHG CH

**FIGURE 4:**
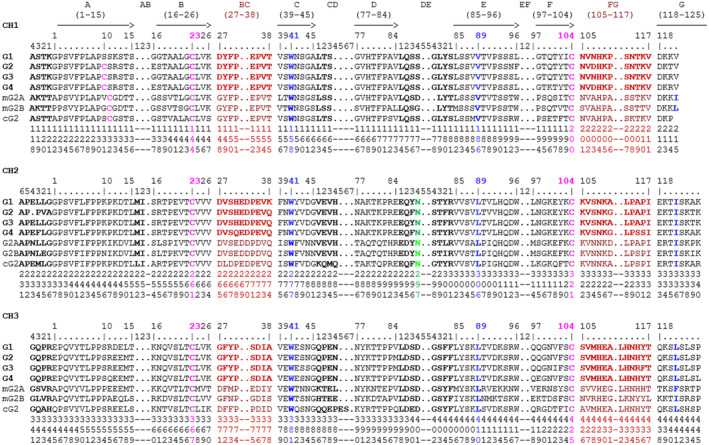
IMGT Protein display of the CH1, CH2, and CH3 of *Homo sapiens* (Homsap) IGHG1*01 (J00228), IGHG2*01 (J00230), IGHG3*01 (X03604), IGHG4*01 (K01316) genes (G1, G2, G3 and G4), *Mus musculus* (Musmus) IGHG2A*01 (V00825) and IGHG2B*01 (V00763), *Canis lupus familiaris* (Canlupfam) IGHG2*01 (IMGT000001) (mG2A, mG2B and cG2, respectively), and the Eu‐IMGT positions^3^ (shown vertically on 3 lines, to be read from top to down), based on the IMGT unique numbering for C domain.^15^ (Drawn by Marie‐Paule Lefranc and Gérard Lefranc, LIGM, Founders and Authors of IMGT® the international ImMunoGeneTics information system®, https://www.imgt.org).

The IMGT Protein displays allow the comparison of several IGHG CH amino acid sequences based on the IMGT unique numbering for C domain.^15^ The IMGT Protein display in Figure 4 comprises the CH1, CH2, and CH3 of the four *Homo sapiens* IGHG genes (alleles*01), those of the *Mus musculus* IGHG2A*01 and IGHG2B*01 and of the *Canis lupus familiaris* IGHG2*01 (species for which Fc‐engineered variants have been described) and the Eu‐IMGT positions,^3^ based on the IMGT unique numbering for C domain.^15^

**FIGURE 5:**
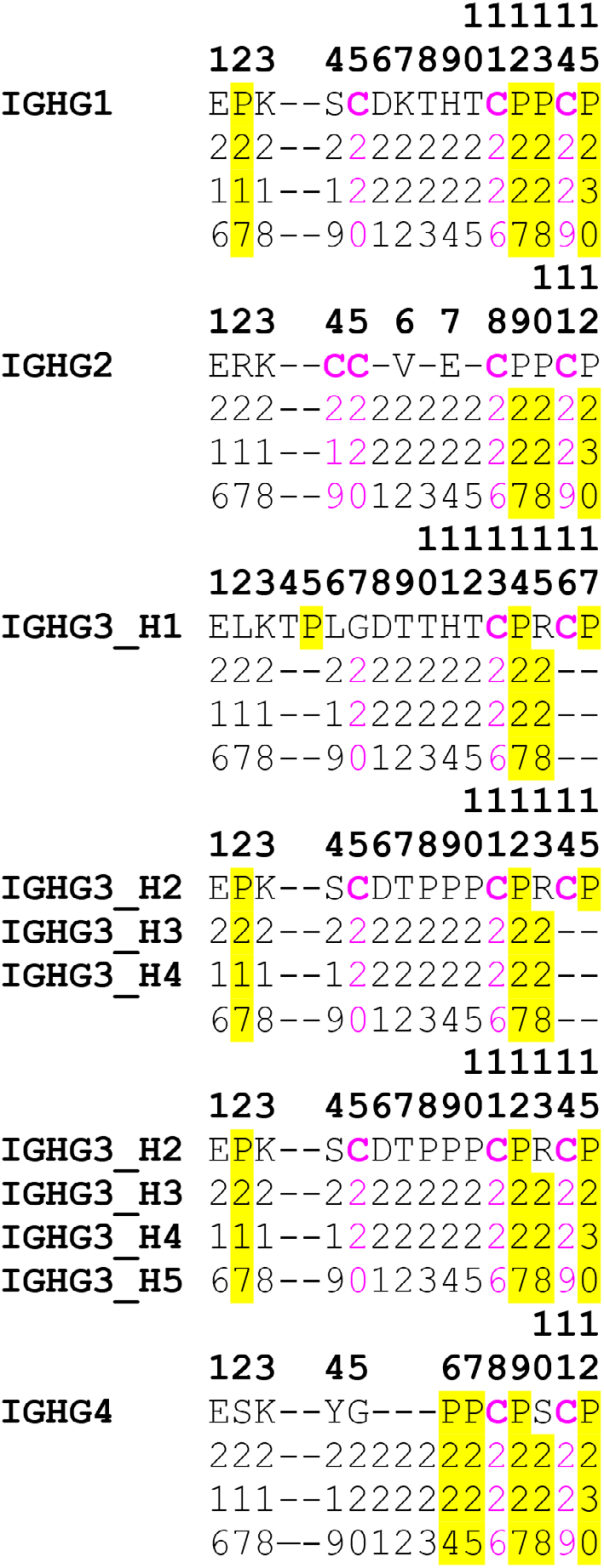
Hinge regions encoded by the hinge exons of the *Homo sapiens* IGHG1, IGHG2, IGHG3, and IGHG4. The hinge IMGT numbering is shown in bold above the sequences (1–15 for IGHG1, 1–12 for IGHG2, 1–17 (H1), and 1–15 (H2, H3, H4, and H5) for IGHG3, 1–12 for IGHG4). (Drawn by Marie‐Paule Lefranc and Gérard Lefranc, LIGM, Founders and Authors of IMGT® the international ImMunoGeneTics information system®, https://www.imgt.org).

The IGHG hinge region (located between CH1 and CH2) is not part of the C domains; it has a length, which varies between the H‐gamma chains of different IgG subclasses and has its own numbering as exemplified in *Homo sapiens* (Figure 5).

## 4 ALLOTYPES

The allotypes have been identified on the constant region of the heavy H‐gamma1, H‐gamma2, H‐gamma3, and H‐alpha2 chains (designated as G1m, G2m, G3m, and A2m allotypes, respectively), and on the light kappa chain (Km allotypes).^54^, ^55^ Gm and Am allotypes have been one of the most powerful tools in population genetics^61^, ^62^, ^63^, ^64^, ^65^, ^66^, ^67^, ^68^ and very instrumental in molecular characterization of the human IGHC genes (gene conversion, multigene deletions in healthy individuals, copy number variations or CNV, and gene order).^10^, ^11^, ^88^, ^89^, ^90^, ^91^, ^92^, ^93^, ^94^, ^95^, ^96^ The extensive polymorphism and the molecular characterization of the allotypes and of the isoallotypes have been reviewed.^54^, ^55^ As the IgG1 is the most frequent subclass used in therapeutic antibodies, a summary of the G1m allotypes and isoallotypes is provided in Table 3, with the IMGT correspondence between the IGHG1 alleles and the G1m alleles and the amino acids encoding the allotypes and isoallotypes.^55^

**TABLE 3:** IMGT correspondence between the IGHG1 alleles and the G1m alleles, and amino acids encoding the allotypes and isoallotypes.^55^ Amino acid positions are according to the IMGT unique numbering for C domain.^15^

IGHG1 alleles	G1m alleles^a^		IMGT amino acid positions^b^								Populations^54^, ^55^
	Allotypes^a^	Isoallotype^c^	CH1		CH3						
			103	120	12	14	101	110	115	116	
				K120 G1m17 R120 nG1m17	D12, L14 G1m1 E12, M14 nG1m1		I101 G1m27	G110 G1m2	R115, Y116 G1m28		
			I103, R120 G1m3^d^								
IGHG1*01, IGHG1*02, IGHG1*05, IGHG1*09	G1m17,1		I	**K**	**D**	**L**	V	A	H	Y	Caucasoid Negroid Mongoloid
IGHG1*03, IGHG1*06	G1m3	*n* *G1m1*, *nG1m17*	**I**	**R**	E	M	V	A	H	Y	Caucasoid
IGHG1*04	G1m17,1,27		I	**K**	**D**	**L**	**I**	A	H	Y	Negroid
IGHG1*07	G1m17,1,2		I	**K**	**D**	**L**	V	**G**	H	Y	Caucasoid Mongoloid
IGHG1*08	G1m3,1	*nG1m17*	**I**	**R**	**D**	**L**	V	A	H	Y	Mongoloid
IGHG1*05p ^e^	G1m17,1,28		I	**K**	**D**	**L**	V	A	**R**	**Y**	Negroid
IGHG1*06p ^e^	G1m17,1,27,28		I	**K**	**D**	**L**	**I**	A	**R**	**Y**	Negroid
IGHG1*09p ^e^	G1m17	*nG1m1*	I	**K**	E	M	V	A	H	Y	Caucasoid
IGHG1*01v	G1m3,1	*nG1m17*	**I**	**R**	**D**	**L**	V	A	H	Y	Engineered
IGHG1*03v	G1m17	*nG1m1*	I	**K**	E	M	V	A	H	Y	Engineered

*Source*: With permission from M‐P. Lefranc and G. Lefranc, LIGM, Founders and Authors of IMGT®, the international ImMunoGeneTics information system®, https://www.imgt.org.

^a^ A Gm allele is defined by its combination of allotypes and isoallotypes. In Negroid populations, the G1m17,1 allele frequently includes G1m27 and/or G1m28, leading to three additional G1m alleles, G1m17,1,27, G1m17,1,28, and G1m17,1,27,28.^54^, ^55^, ^63^ The G1m17,1,27 allele confirmed by mass spectrometry has been assigned to the IGHG1*04 allele.^54^, ^55^, ^58^

^b^ Amino acids positions are according to the IMGT unique numbering for C domain.^15^ In the table, amino acids corresponding to G1m allotypes are shown in bold. Allotypes on CH1 include CH1 G1m17 K120 (pale blue background color) versus CH1 nG1m17, R120 which is also G1m3 (dark blue) in IGHG1 owing to the presence of I103.^54^, ^55^ Allotypes on CH3 include CH3 G1m1 D12, L14 (yellow) versus the isoallotype nG1m1 E12 M14 (pale yellow), CH3 G1m27 I101 (green), CH3 G1m2 G110 (purple), and CH3 G1m28 R115, Y116 (orange).

^c^ The isoallotype nG1m1 (CH3 E12 M14) which is present on the Gm1‐negative H‐gamma1 (and on the G2, G3, and G4 chains) and the isoallotype nG1m17 (CH1 R120), which is present on the Gm17‐negative H‐gamma1 chains (and on the G3 and G4 chains)^54^, ^55^ (Figure 3C,D) are shown in italics.

^d^ The presence of R120 is detected by anti‐nG1m17 antibodies, whereas the simultaneous presence of I103 and R120 on the H‐gamma1 chains is detected by anti‐Gm3 antibodies.^54^, ^55^

^e^ IGHG1*05p, IGHG1*06p, and IGHG1*09p amino acids are expected,^54^, ^55^ but not yet sequenced at the nucleotide level, and therefore, these alleles are not shown in IMGT Repertoire (IG and TR) Alignments of alleles. The definitive allele number will be assigned by chronological order following the availability of the nucleotide sequences.

The amino acids encoding all known allotypes and isoallotypes have been characterized,^54^, ^55^ which permits the current assignment of allotypes and isoallotypes to therapeutic antibodies, based on chain aa sequences^9^, ^56^, ^57^ and liquid chromatography–mass spectrometry (LC–MS) results.^58^, ^59^, ^60^ It is the presence of a leucine (instead of the usual valine) on a peptide obtained from a LC–MS screening for Fc allelic variants^58^ and previously unambiguously identified as the leucine characteristic of the G1m27 allotype^54^, ^55^ that led to the assignment of the IGHG1*04 allele to the G1m17,1,27 allele which was detected serologically (Table 3).

The most frequent G1m alleles used in therapeutic antibodies are G1m17,1 and G1m3 (Table 3). G1m17,1 is found, in populations, on several IGHG1 alleles (*01, *02, *05, *09), which only differ between them at the nucleotide level (Table 3). The percentage of identity, provided in the description of therapeutic antibodies, is by reference to the *Homo sapiens* IGHG1*01 aa sequence (e.g., 100%). The G1m17,1 allele comprises CH1 K120 (G1m17) and CH3 D12, L14 (G1m1) (“KDL”) (Table 3, Figure 3A). G1m3 is found, in populations, on the IGHG1*03 and IGHG1*06 alleles (which only differ between them by 1 nt) (Table 3). The percentage of identity, provided in the description of therapeutic antibodies, is by reference to the *Homo sapiens* IGHG1*03 aa sequence (e.g., 100%). The G1m3, nG1m1 allele comprises CH1 R120 (G1m3 expressed with I103, only present on IGHG1) (Table 3) and CH3 E12, M14 (nG1m1) (“REM”).^3^

Two engineered G1m variants are used in therapeutic antibodies, IGHG1*03v and IGHG1*01v (Table 3).^55^ The first one, IGHG1*03v is G1m3>G1m17, nG1m1 (CH1 R120>K, CH3 E12, M14) (“KEM”), engineered to decrease the immunogenicity of the G1m3 IGHG1*03 H‐gamma1 chain by replacing the R120 (G1m3) with K120 (G1m17). The second one, IGHG1*01v, G1m3, G1m1 (CH1 K120>R, CH3 D12, L14) (“RDL”), has the same allotypes as the natural G1m3,1 allele assigned to the gene allele IGHG1*08. However, IGHG engineered variants have their own nomenclature. Indeed, they cannot be assigned to the name of natural IGHG alleles which are defined at the nucleotide level.^3^

The constant region of the H‐gamma2 chain is frequently encoded by the *Homo sapiens* IGHG2*01 (100%), G2m.. (CH2 V45.1) (the two dots indicate the absence of the G2m23 allotype^54^, ^55^).^3^ CH2 V45.1 is in the CD transversal strand (Figure 3B). There is one engineered variant, IGHG2*02v, G2m23>G2m.. (CH2 M45.1>V) (for a less immunogenic H‐gamma2 chain). The H‐gamma4 chain has no allotype but expresses a non‐allotype, either the nG4m(a) CH2 L92 encoded by IGHG4*01 (100%) (Figure 3D), also encoded by IGHG1*01 (Figure 3A) and IGHG3*01 (Figure 3C), or the nG4m(b) CH2 V92 encoded by IGHG4*02 (100%), also encoded by IGHG2*01 (Figure 3B).^3^

The H‐gamma3 chain has been so far rarely used in therapeutic antibodies owing to its high allelic diversity and long polymorphic hinge (62 aa for the IGHG3 with a 4‐exon h1‐h4 hinge^97^ [IGHG3*01 (X03604, G3m5* (G3m5,10,11,13,14,26,27), from which an IGHG3‐specific 4‐exon hinge probe^98^ (X04646) has been isolated], 47 aa for the IGHG3 with a 3‐exon h1,h3,h4 hinge^99^ [IGHG3*03 (X16110, G3m24* (G3m5,6,11,24,26)], 32 aa for the IGHG3 with a 2‐exon hinge^100^ [IGHG3*4 (X99549, G3m5,10,11,13,14)]).^3^ However, there is a growing interest for IGHG3 in therapeutic antibody engineering owing to the H‐gamma3 effector properties and an example of the H‐gamma3 constant region with a 4‐exon hinge, encoded by the *Homo sapiens* IGHG3*01, G3m5* (G3m5,10,11,13,14,26,27) CH3 S44, M84, Q98, I101, R115, and F116 (Figure 3C) has been used in a therapeutical antibody. The IMGT G3m allele butterfly representation provides a graphical overview of the six major G3m alleles, characterized by the association of several allotypes on the same H‐gamma3 chain.^54^, ^55^ The coding region of the IGHGP gene, a pseudogene owing to the lack of a switch region, has not been tested although it shows no other major defect.^101^

Allotypes have been characterized on the L‐kappa chains (but not on the L‐lambda chains which instead differ by their isotypes, as there are several functional IGLC genes).^3^, ^10^ The *Homo sapiens* IGKC*01 (100%) Km3 A45.1, V101 is the most extensively used Km allele in therapeutic antibodies, being the most represented in populations and the less immunogenic.^3^, ^10^, ^54^, ^55^

## 5 FC IGHG ENGINEERED VARIANTS

### 5.1 Therapeutic antibody effector properties and half‐life

Whereas the antibody variable domains are frequently engineered for specificity, affinity, and humanization,^30^, ^41^, ^102^, ^103^, ^104^, ^105^, ^106^, ^107^, ^108^, ^109^, ^110^, ^111^, ^112^ the antibody constant regions of the heavy chains are frequently engineered to modify the effector properties of the therapeutic antibodies.^69^, ^70^ Amino acids changes of the IGHG CH domains are engineered at positions involved (i) in ADCC mediated by NK cells through the Fc binding to FCGR3A (FcgammaRIIIa, CD16a), (ii) in ADCP mediated by phagocytic cells (macrophages, monocytes, and neutrophils) through the Fc binding to their activatory FCGR (FcgammaR), (iii) in B‐cell inhibition by coengagement of antigen and inhibitory FCGR2B (FcgammaRIIb) on the same cell, (iv) in CDC mediated by the Fc binding to the first component of complement C1q, and (v) in half‐life mediated by Fc binding to FCGRT (FcRn).^69^, ^70^ Little work has been done so far to modify the Fc binding to FCGR1A (FcgammaRI), the high affinity for IgG, considered as being saturated by IgG in circulation; however, there is a recent interest for understanding its function.

### 5.2 Standardized IMGT‐engineered variant names

The standardized IMGT‐engineered variant nomenclature has been set up for an easier comparison between engineered antibody variants involved in effector properties (ADCC, ADCP, and CDC) and half‐life of therapeutic antibodies (Table 4). The IMGT nomenclature also includes structure variants, involved in the format of therapeutical antibodies.^71^ Only a few of them related to effector variants are included in Table 4 (highlighted in yellow). The property and function type is indicated by a number from 1 to 18. A pink background highlights the ADCC reduction (1), CDC reduction (5), ADCC and CDC reduction (6), FCGR2B binding increase and B‐cell inhibition (7), and ADCC reduction owing to knock out of CH2 84.4 glycosylation (8). A pale green background highlights the ADCC enhancement (2), ADCC and ADCP enhancement (3), and CDC enhancement (4). A blue background highlights the half‐life increase or decrease (9). A pale orange background highlights the physicochemical properties (e.g., abrogation of binding to Protein A, thermal stability, pI, reduced acid‐induced aggregation) (10). Only a few examples of structure variants (which comprise (11) to (18)^71^) are given in Table 4, highlighted in yellow, for additional disulfide bridge for domain stabilization (11), prevention of IgG4 half‐IG exchange (12), hexamerization (13), and control of half‐IG exchange of bispecific IgG (18).

**TABLE 4:** IMGT‐engineered Fc variants of therapeutical antibodies and IMGT topological motifs.

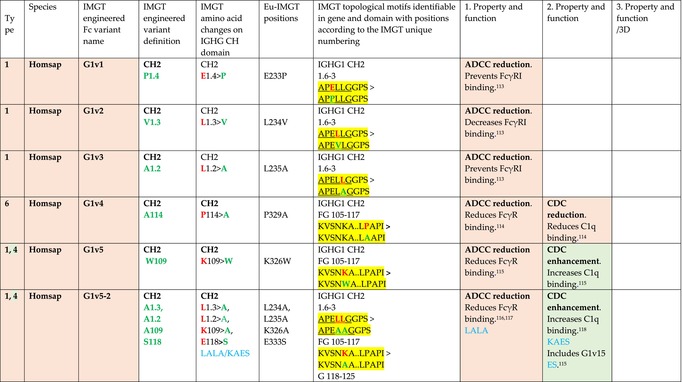
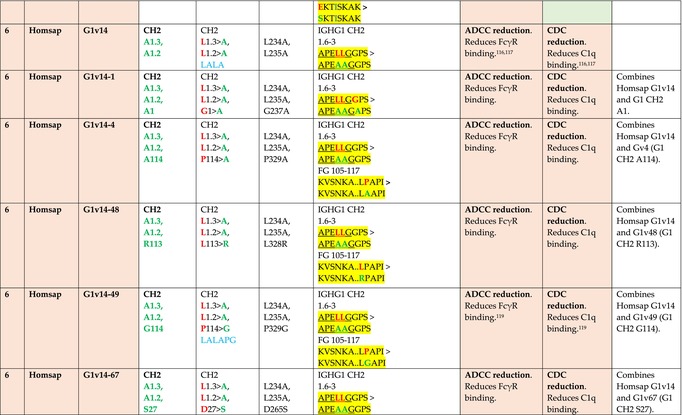
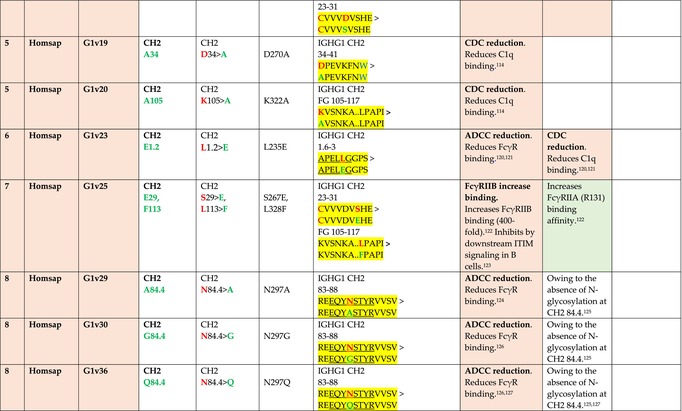
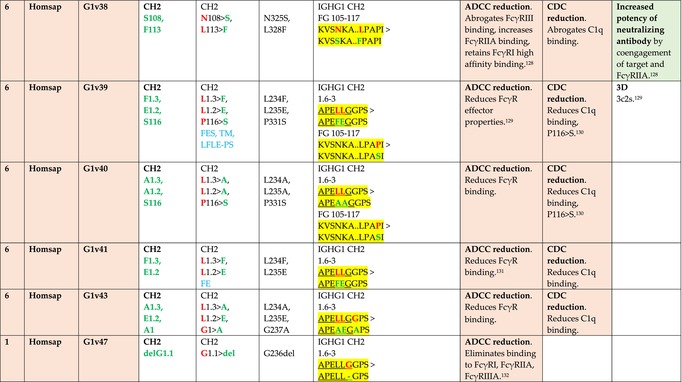
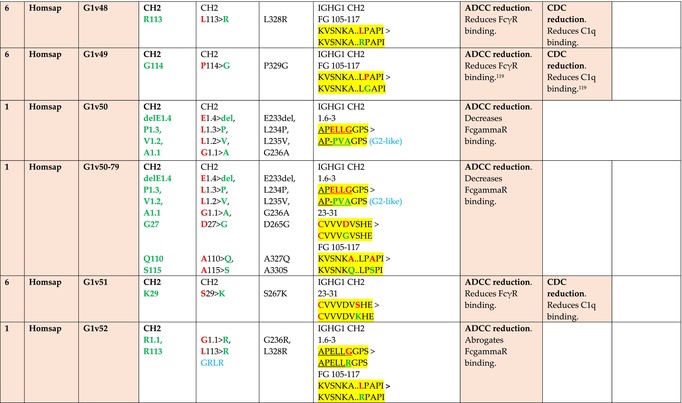
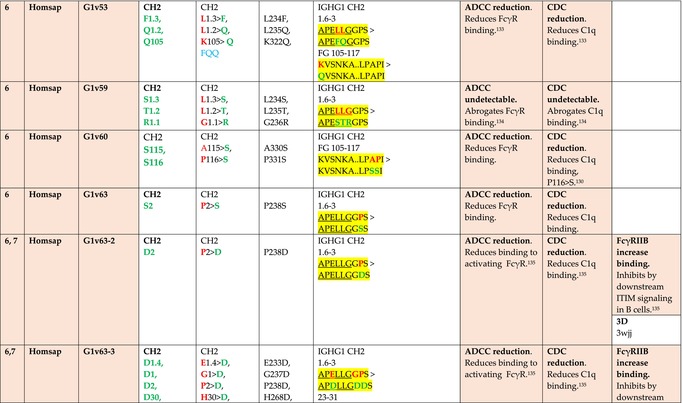
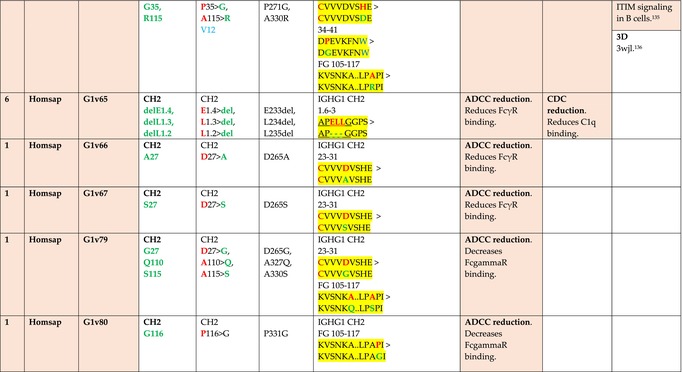
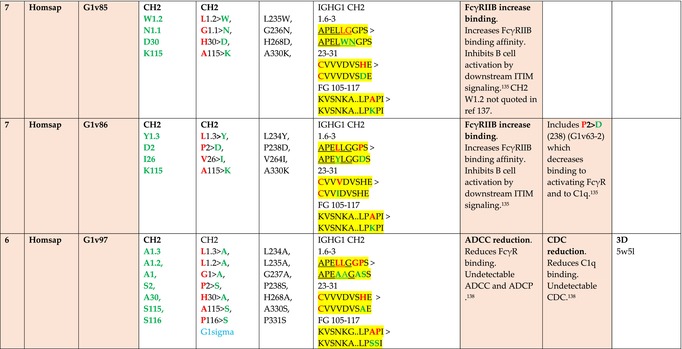
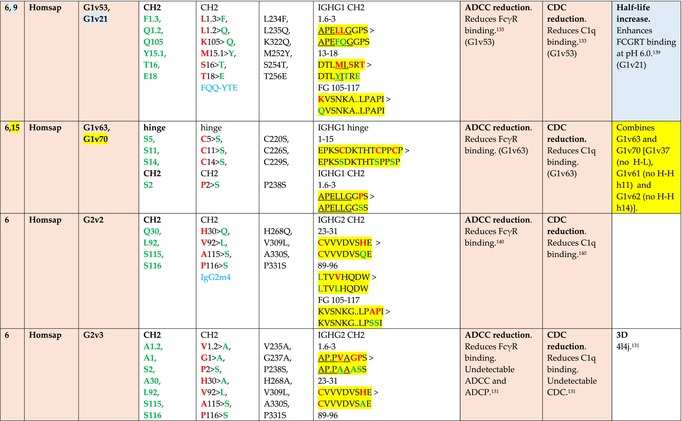
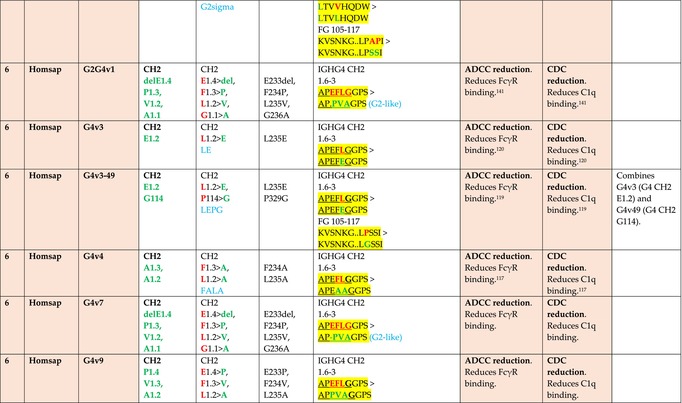
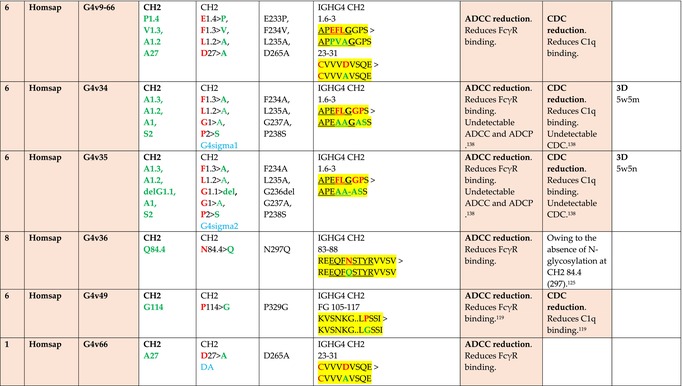
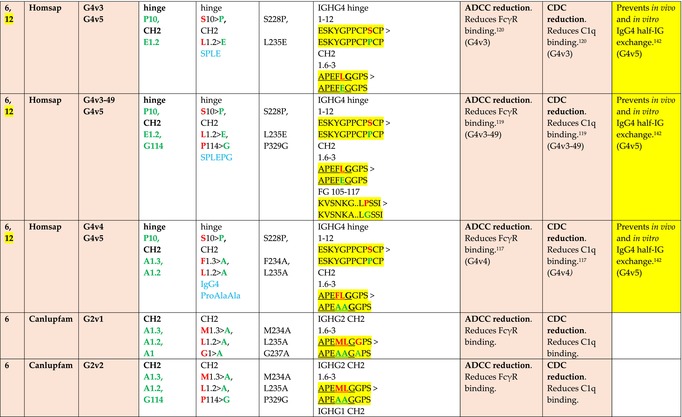
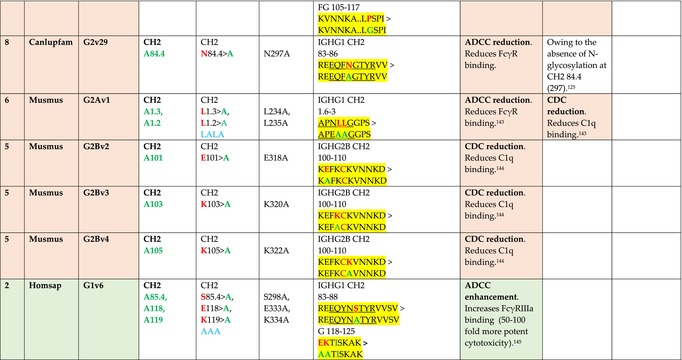
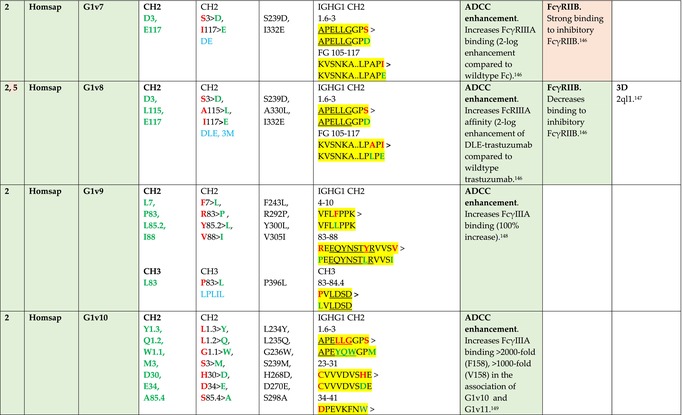
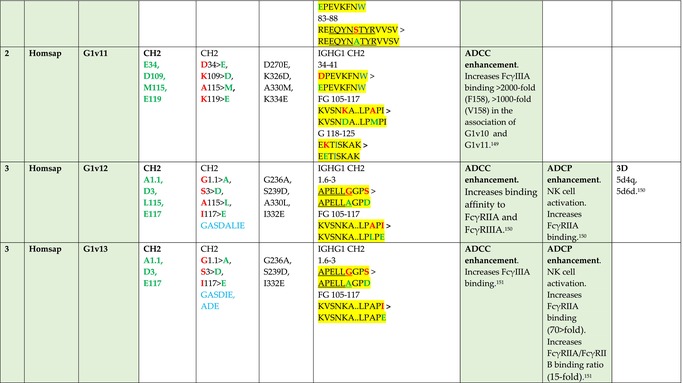
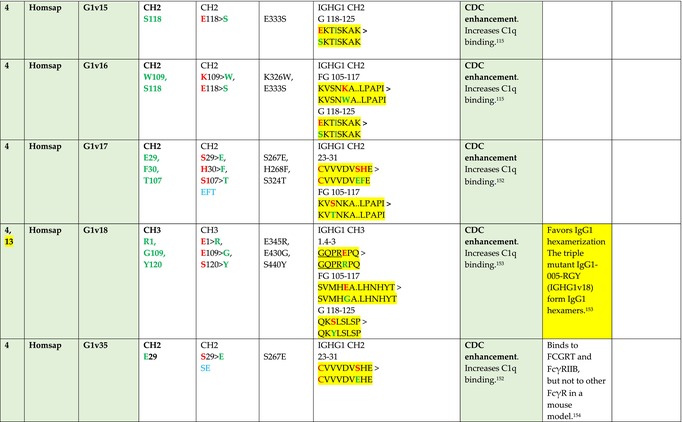
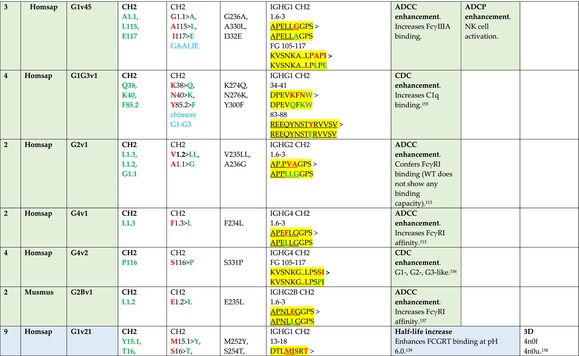
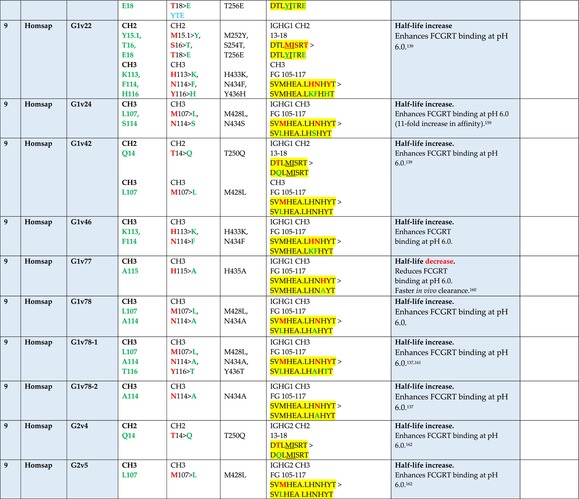
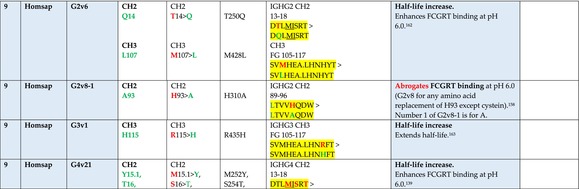
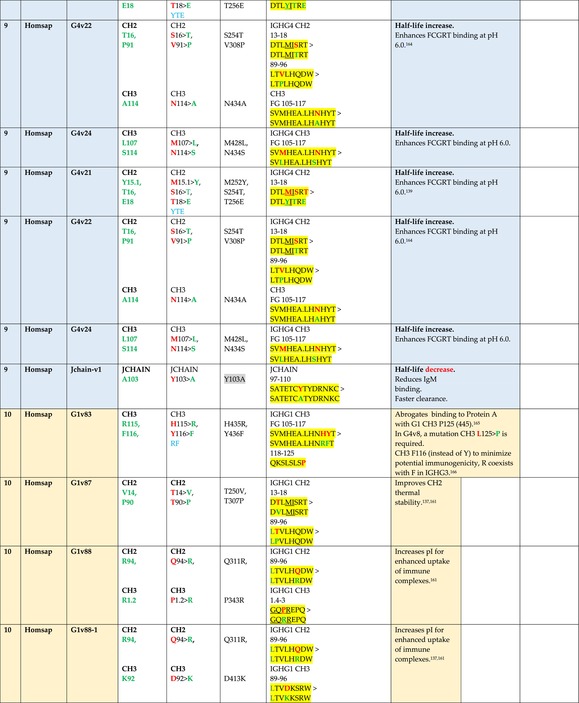
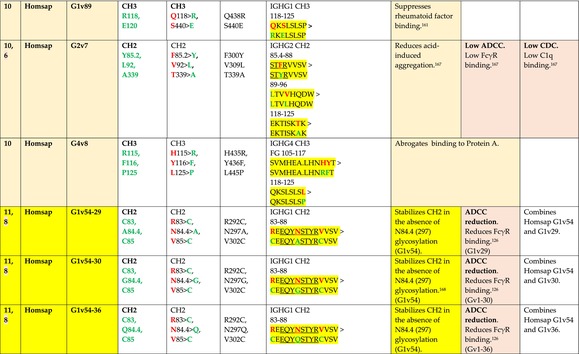
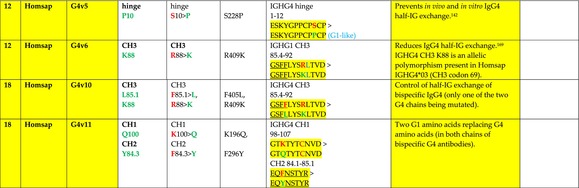

*Note*: Amino acids are shown in the one‐letter abbreviation.^21^ Amino acid positions of the constant regions are according to the IMGT unique numbering for C domain.^15^ IMGT Scientific chart https://www.imgt.org/IMGTScientificChart/Numbering/Hu_IGHGnber.html (e.g., E233P [Eu‐IMGT] is CH2 E1.4>P and the engineered amino acid is CH2 P1.4 [IMGT unique numbering for C domain]).^15^ The IMGT topological motifs are highlighted in yellow and shown with the engineered amino acid changes are in bold (red before the change, green after the change. Amino acids of the motifs at additional positions in the IMGT unique numbering for C domain^15^ (by comparison to the IMGT unique numbering for V domain^14^) are underlined. Alias variant names found in the literature are written in blue in column 5 “IMGT amino acid changes on IGHG CH domain.” The background color indicates a reduction (pink color) or an enhancement (green color) of the involved “Property and function.” For other “Property and function,” background colors refer to half‐life (pale blue color), physicochemical properties (pale orange), or structure (yellow). The property and function type is indicated by a number from 1 to 18. (1) Antibody‐dependent cellular cytotoxicity (ADCC) reduction. (2) Antibody‐dependent cellular cytotoxicity (ADCC) enhancement. (3) Antibody‐dependent cellular cytotoxicity (ADCC) and antibody‐dependent cellular phagocytosis (ADCP) enhancement. (4) Complement‐dependent cytotoxicity (CDC) enhancement. (5) Complement‐dependent cytotoxicity (CDC) reduction. (6) Antibody‐dependent cellular cytotoxicity (ADCC) and complement‐dependent cytotoxicity (CDC) reduction. (7) FCGR2B binding increase and B‐cell inhibition (coengagement of antigen and FcγR on same cell). (8) Knock out CH2 84.4 glycosylation. (9) Half‐life increase or decrease. (10) physicochemical properties (e.g., abrogation of binding to Protein A, thermal stability, pI, reduced acid‐induced aggregation). Examples are only given for the sections. (11) Additional disulfide bridge for domain stabilization, including scFv. (12) Prevention of IgG4 half‐IG exchange. (13) Hexamerization (G1v18 in 4.). (18) Control of half‐IG exchange of bispecific IgG.

The IMGT‐engineered Fc variant name^71^ comprises species and gene name (abbreviated), the letter “v” with a number (e.g., Homsap G1v2). The IMGT variant definition includes, per domain (e.g., CH2), the amino acid(s) of the variant in the one‐letter abbreviation^21^ with its position in the IMGT unique numbering for C domain,^15^ for example, Homsap G1v2 CH2 V1.3 (Table 4).

The IMGT variant characterization displays: (i) the IMGT aa change(s) per CH domain with CH position (letter in red before the change, green after the change) (e.g., CH2 L1.3>V), (ii) aa change(s) with Eu‐IMGT position^3^ (e.g., L234V) (considered as a ruler independent of the Eu aa owing to ambiguities in the Eu sequence^170^), (iii) the IMGT motif with gene name, CH domain and CH positions according to the IMGT unique numbering,^15^ (e.g., IGHG1 CH2 1.6–3). The sequence of the motif is shown with the aa involved in the change in bold and highlighted, red before the change, and green after the change (e.g., APE **L** LGGPS > APE **V** LGGPS), and (iv) information from the literature regarding “property and function.”^113^, ^114^, ^115^, ^116^, ^117^, ^118^, ^119^, ^120^, ^121^, ^122^, ^123^, ^124^, ^125^, ^126^, ^127^, ^128^, ^129^, ^130^, ^131^, ^132^, ^133^, ^134^, ^135^, ^136^, ^137^, ^138^, ^139^, ^140^, ^141^, ^142^, ^143^, ^144^, ^145^, ^146^, ^147^, ^148^, ^149^, ^150^, ^151^, ^152^, ^153^, ^154^, ^155^, ^156^, ^157^, ^158^, ^159^, ^160^, ^161^, ^162^, ^163^, ^164^, ^165^, ^166^, ^167^, ^168^, ^169^

### 5.3 Standardized IMGT topological motifs

Amino acid changes in engineered Fc antibodies are from publications and from sequences published at WHO INN MedNet.^81^, ^82^ The lengths of the motifs reported in Table 4 have been standardized to correspond, for a given domain (e.g., CH2), to a given label (e.g., FG) or to a given length (e.g., 1.6–3), in order to facilitate visually the identification of the involved aa in a structural feature (strand, A‐extn, turn, or loop) of the chain sequence. These motifs defined based on the IMGT unique numbering for C domain are the IMGT topological motifs (Table 5).

**TABLE 5:** IMGT topological motifs involved with engineered variants of the IGHG. (A) Reduction in effector properties (types 1, 5 to 8). (B) Enhancement of effector properties (types 2 to 4). (C) Half‐life increase or decrease (type 9). (D) Physicochemical properties (type 10).

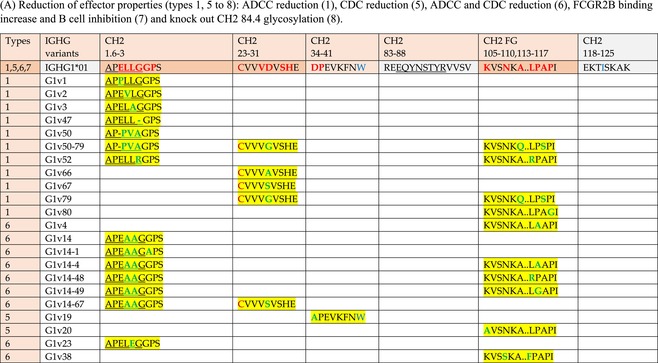
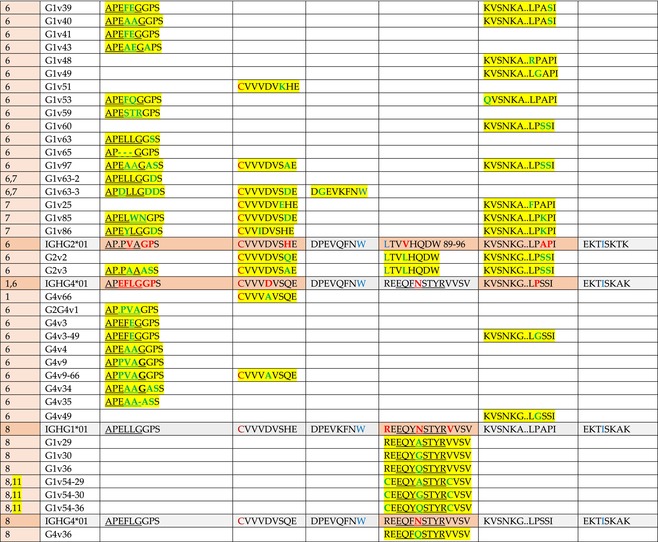
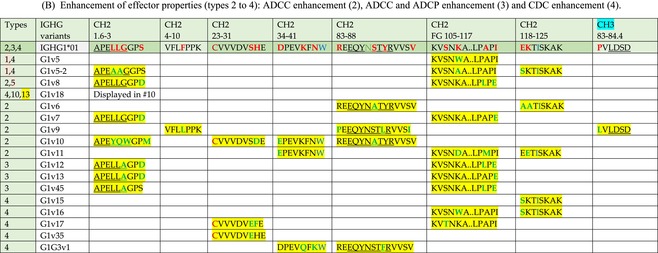
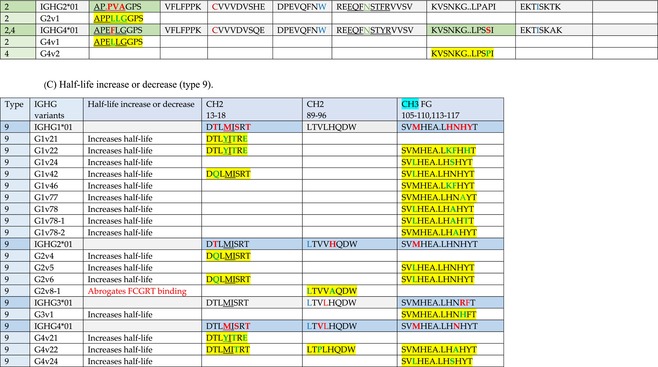
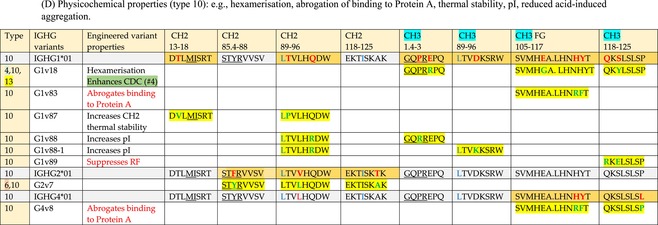

*Note*: Letters in red indicate amino acid changes identified in Fc‐engineered variants.

Three IMGT topological motifs characterize the Fc‐engineered variants of effector properties. They are localized on the CH2 of the IGHG chains, as expected. The first motif CH2 1.6–3 (9 aa) corresponds to “the lower hinge” in literature (Table 5). Actually, it comprises the extension A‐extn (1.6 to 1.1) of the CH2 A‐strand and the first three aa GPS (1–3) of the basic A‐strand (Table 1). Underlined aa in the motif correspond to the additional positions in the IMGT unique numbering for C domain,^15^ for example, in CH2 1.6–3, APELLGGPS (IGHG1*01), AP.PVAGPS (IGHG2*01), and APEFLGGPS (IGHG4*01), which correspond to 1.6, 1.5, 1.4, 1.3, 1.2, and 1.1, respectively. In the A‐extn of the CH2 of IGHG2, the gap is at 1.4 by alignment comparison of the IGHG translations (Figure 4),^3^, ^15^ including that of IGHGP (not shown),^101^ and corresponds to the deletion of CH2 E1.4.^3^, ^10^ The second IMGT motif, CH2 23‐BC‐41 (17 aa), delimited by C23 and W41, includes BC 27–31, 34–38. It is worth to note that the CH2 BC loop is 10 aa long and has two unoccupied positions 32–33 (whereas the CH1 and CH3 BC are shorter with 8 aa and four unoccupied positions 31–35) (Figure 3) (Table 1). The third IMGT motif for Fc effector properties is the CH2 FG loop 105–110, 113–117 (11 aa).

Three IMGT topological motifs characterize the Fc‐engineered variants for half‐life, two are localized on the CH2 of the IGHG chains, and the third one on the CH3, as expected. The first IMGT motif for half‐life is CH2 13–18. The motif sequence for the four *Homo sapiens* IGHG is DTLMISRT. It includes the AB turn with the two additional positions M15.1 and I15.2 (Tables 1 and 5). The second motif for half‐life is IGHG CH2 89–96 with H93 which needs to be preserved in the motif sequence (LTVLHQDW for IGHG1, IGHG3 and IGHG4, and LTVVHQDW for IGHG2) for half‐life increase. Indeed, the variant G2v8–1 A93 (as well as any variant G2v8 with any other aa replacement of H93 except cystein) abrogates FCGRT binding at pH 6.0 (Table 4). The third motif for half‐life is localized on the CH3 domain. The motif is CH3 FG loop 105–110, 112–117 (12 aa) (Table 5).

## 6 CONCLUSION

The advances of the therapeutic antibodies are a constant source of discovery in therapy and in fundamental biology. Standardized sequence and structure analysis using the IMGT databases, tools, and web resources and the IMGT Scientific chart rules have contributed to bridge genes, sequences, and structures, with applications in the field of the therapeutic antibodies and immunotherapy, for the V‐DOMAIN of the IG and TR (standardized definition of the CDR‐IMGT, humanization and antibody engineering, repertoire, and specificity analysis).^30^, ^41^, ^102^, ^103^, ^104^, ^105^, ^106^, ^107^, ^108^, ^109^, ^110^, ^111^, ^112^ The IMGT nomenclature of the allotypes and the identification of the involved amino acids,^54^, ^55^ the IMGT nomenclature of the Fc‐engineered variants^71^ (Table 4) and the characterization of the IMGT topological motifs (Table 5) represent the contributions of the IMGT Scientific chart rules to bridge sequences and structures for the C‐DOMAIN of the IG in the interactions Fc‐FcgammaR, complement and other proteins.

Indeed, as shown in Table 5, a topological motif may participate, depending on the aa changes to which it is associated (in the same motif or in another motif), to reducing (pink background, types 1, 5, to 8) or to enhancing (green background, types 2 to 4) effector properties. The topological motif IGHG CH2 83–88 (e.g., REEQYNSTYRVVSV for IGHG1, REEQFNSTYRVVSV for IGHG4) that contains the N84.4 site of glycosylation is an example. The absence of glycosylation in the G1v29 A84.4, G1v30 G84.4, G1v36 Q84.4, and G4v36 Q84.4 variants reduces ADCC. In contrast, the variant G1v6 CH2 A85.5, A118, and A119, characterized by the presence of A85.5 (in the motif CH2 83–88 where N84.4 is conserved), associated with A118 and A119 (which belongs to the CH2 G‐strand 118–125 motif) increases FCGR3A (FcγRIIIa) binding and results in ADCC enhancement (Table 4).

The IMGT topological motifs may contribute to the distinction between types. As an example, the variant G1v18 was first classified among type 4 (CDC enhancement) and format type 13 (hexamerization). However G1v18 did not share the same IMGT motifs as the other variants of type 4 (CH2 BC and FG) but instead, three IMGT motifs located on CH3 (1.4–3, FG, G‐strand 118–125) as observed in variants of type 10 (physicochemical properties). This highlights that the CDC enhancement was due to interactions between Fc leading to the hexomerization, justifying that G1v18 (4,10,13) motif be displayed in type 10.

Thus, the IMGT topological motifs may throw light on predominant interactions in comparison with variants of the same or other types. They represent working tools, based on a standardized description of the engineered variants, for an integration of the information provided by the sequences (genes and alleles), crystallographic data (structures) and experimental data (functions). By providing a common language, the IMGT unique numbering is key for future high‐throughput computing analysis.

## CONFLICT OF INTEREST STATEMENT

No conflict of interest to declare.

## Data Availability

All data described in this manuscript have been published previously or are included in Tables 4 and 5.
